# Association of Moderate Coffee Intake with Self-Reported Diabetes among Urban Brazilians

**DOI:** 10.3390/ijerph8083216

**Published:** 2011-08-03

**Authors:** Liliane M. M. Machado, Teresa H. M. da Costa, Eduardo F. da Silva, José G. Dórea

**Affiliations:** 1 Núcleo de Nutrição, Laboratório de Bioquímica da Nutrição, Faculdade de Ciências da Saúde, Universidade de Brasília, Campus Universitário Darcy Ribeiro-Asa Norte. CEP 70910-900 Brasília-DF, Brazil; E-Mails: liliane.machado25@gmail.com (L.M.M.M.); dorea@rudah.com.br (J.G.D.); 2 Departamento de Estatística, Universidade de Brasília, Instituto de Ciências Exatas, Campus Universitário Darcy Ribeiro-Asa Norte. CEP 70910-900 Brasília-DF, Brazil; E-Mail: edufrei@unb.br

**Keywords:** coffee intake, diabetes mellitus, chlorogenic acids, caffeine, body mass index

## Abstract

Coffee has been associated with reductions in the risk of non-communicable chronic diseases (NCCD), including diabetes mellitus. Because differences in food habits are recognizable modifying factors in the epidemiology of diabetes, we studied the association of coffee consumption with type-2 diabetes in a sample of the adult population of the Federal District, Brazil. This cross-sectional study was conducted by telephone interview (n = 1,440). A multivariate analysis was run controlling for socio-behavioural variables, obesity and family antecedents of NCCD. A hierarchical linear regression model and a Poisson regression were used to verify association of type-2 diabetes and coffee intake. The independent variables which remained in the final model, following the hierarchical inclusion levels, were: first level—age and marital status; second level—diabetes and dyslipidaemias in antecedents; third level—cigarette smoking, supplement intake, body mass index; and fourth level—coffee intake (≤100 mL/d, 101 to 400 mL/day, and >400 mL/day). After adjusting hierarchically for the confounding variables, consumers of 100 to 400 mL of coffee/day had a 2.7% higher (p = 0.04) prevalence of not having diabetes than those who drank less than 100 mL of coffee/day. Compared to coffee intake of ≤100 mL/day, adults consuming >400 mL of coffee/day showed no statistically significant difference in the prevalence of diabetes. Thus, moderate coffee intake is favourably associated with self-reported type-2 diabetes in the studied population. This is the first study to show a relationship between coffee drinking and diabetes in a Brazilian population.

## 1. Introduction

Coffee is one of the most widely consumed beverages in the Western world. Therefore, concerns about health risks and benefits associated with coffee (and attendant caffeine intake) shown by epidemiological studies are likely to be influenced by social and metabolic profiles of studied populations. Coffee, in its beverage form, is a complex chemical mixture, especially after the roasting process [1]; however, because it is rich in antioxidants and nutrients it generally seems to be associated with a healthy profile in consumers, and studies have shown that coffee can be considered a functional food [2]. Indeed, regular coffee drinking (depending on the quantity consumed) can directly affect the intake of micronutrients (K, Mg, Mn, Cr, niacin) and antioxidant substances. It has also shown positive effects on various aspects of health, such as neurological (infant hyperactivity, Alzheimer’s and Parkinson’s diseases) and metabolic disorders (diabetes mellitus, gallstones, liver cirrhosis), and gonadal and liver function [2].

Although coffee is a beverage rich in antioxidants, it is also rich in caffeine, which is the most studied compound of coffee. This methylxanthine increases attention and alertness levels, and improves work performance and readiness for physical activity [3]. However, caffeine may also impair insulin sensitivity and glucose tolerance [4,5].

The epidemiology of diabetes mellitus has increased substantially in developed countries and is now also becoming a major problem in developing countries [6]. Indeed, the epidemiology of diabetes mellitus in Brazil has substantially changed in the last decade [7,8], and projections for 2030 suggest that 11.3 million people will be affected [7]. The metabolism underlying type-2 diabetes poses a greater risk for hypertension, dyslipidaemia, and cardiovascular diseases; therefore, diet and nutrition are central to controlling and preventing NCCD [6]. Because coffee drinking impacts a broad demographic, earlier findings that it might be associated with NCCD such as diabetes mellitus have raised interest in epidemiological studies in Eastern and Western industrialized countries. Thus, both prospective [9–13] and cross-sectional [14,15] studies indicate a decreased risk of type-2 diabetes associated with coffee drinking.

The factors associated with coffee intake and risk of type-2 diabetes are likely to depend on variables not only intrinsically related to coffee preparation and consumption [16,17], but also to other environmental and dietary variables [14,18,19], such as smoking, sedentarism and poor diet. Thus, we sought to evaluate the association of coffee consumption and self-reported type-2 diabetes in a sample of urban adults living in the Federal District, Brazil.

## 2. Experimental Section

The Federal District of Brazil consists of the country’s capital (Brasília) and several other satellite towns and communities covering an area of 5,801.9 km^2^ and with a population of 2.3 million people. We designed a cross-sectional population-based survey with adult subjects living in the Federal District. The study was conducted through a simple-standardized questionnaire validated in a pilot study performed before the research began. The questionnaire was based on the Behavioral Risk Factor Surveillance System (BRFSS) [20], which has been previously validated [21]. Through telephone interview we collected information on detailed coffee intake, socio-behavioural aspects, years of schooling, physical activity, cigarette smoking, alcohol intake, self-reported weight and height, use of nutritional supplements, and family and personal history of NCCD. Self-reported weight and height have been previously validated in Brazil [22,23] The questions used to assess diabetes mellitus presence, according to the BRFSS [20] were: “*Did your doctor say that you have diabetes?”* and *“What is the name of the medicine you take to control diabetes?”* If the interviewed person had diabetes diagnose, questions were asked to define the diabetes type: *“Did your doctor say which type of diabetes you have?”* and “*When your diabetes diagnosis was made how old were you?”* and also *“Do you use insulin injections every day?”*

All questionnaires were reviewed by one of the authors (L.M.); all information collected about other NCCDs was obtained by asking if the person had received the diagnosis from a doctor or health professional. Inclusion criteria were age ≥18 years and agreement to participate in the study. For mobile telephones the owner was contacted and for residential telephones a permanent resident was invited to enter the study. In the case of two phone lines for the same address or individual the first interview was kept, the telephone number excluded and the following selected number contacted. Exclusion criteria included <18 years of age, incomplete data in the questionnaire and any person who did not agree with the term of consent. When a number was called more than ten times and we received no answer or only a standard recorded message from the phone company, that number was excluded. The total number of calls was calculated to be 1,400 following a sampling protocol.

This study was conducted according to the guidelines laid down in the Declaration of Helsinki and all procedures involving human subjects were approved by the Research Ethics Committee of the University of Brasília Faculty of Health Sciences (020/2006). The participants were properly informed of the objectives of the study and oral informed consent was obtained from all of them; consent was recorded electronically.

### 2.1. Sampling

The individuals selected for interview were randomly drawn from mobile and residential telephone lists. Due to the high cost of mobile telephone calls, we expected that 80% of the sample would be from residential numbers. For sample calculation, a coffee intake prevalence of 91% was considered. Precision of 1.5% and a significance level of 5% were adopted and a sample of 1,400 subjects was obtained. The telephone interview was conducted by a team of trained nutrition students. The interview was recorded electronically (PCTEL version 1.0, Brasília, Brazil) and the questionnaire answers were recorded directly into a computer using Epi Info version 6.04d (CDC, USA).

### 2.2. Statistical Analysis

SPSS version 13 (for Windows) was used to perform descriptive statistical analyses. The Fisher exact test was run to ascertain statistical differences between categorical variables (categories of coffee intake with sex, age, years of schooling, marital status, alcohol and supplement use, cigarette smoking, physical activity practised, family disease history). The Poisson regression model was employed because the outcome variable (type-2 diabetes status; yes, 4% and no 96% prevalence), is binary, and the study is cross-sectional [24]. Sample design was robust to reach people from all regions of the Federal District. Percentages of population in the main zones from the census (http://www.brasilia.df.gov.br/) and from the sample were, respectively: zone 1, 1.2% and 1.8%; zone 2, 2.5% and 1.6%; zone 3, 6.5%–5.0%, and zone 4, 13.7% and 12%. Due to the large number of women interviewed at the expense of men, sample weighting was performed. The values of variables were weighted for sex, age and years of schooling; the weights were determined by the ratio between the proportions of the Census from the year 2000 [25] and the sample (Supplemental Data).

Coffee intake was obtained as a continuous variable (mL/day) and was tested for trend with no statistical significance. For this reason coffee intake was categorized and included in the model as a categorical variable. We used the STATA (version 10) program to run Poisson regression, using a robust variance procedure, in a univariate and a multivariate analysis to obtain prevalence ratios (PR) and 95% confidence intervals (95% CI). Multivariate analysis was performed using a hierarchical linear regression model. The variables were introduced according to their causality for type-2 diabetes and coffee intake.

In the multivariate model variables were adjusted to those of the same level (explanatory variables) and higher levels (confounders), *i.e.*, from level one to level four. Each variable block level was included sequentially and the variables with p ≤ 0.20 in the likelihood ratio test remained in the model. The first level of the hierarchical linear regression model was composed of the socio-demographic variables: sex, age, years of study and marital status; the second level by history of family chronic diseases: hypertension, cancer, Parkinson’s disease, Alzheimer’s disease, diabetes and dyslipidaemias; the third level was made up of body mass index (BMI in Kg/m^2^) and lifestyle characteristics related to cigarette smoking, use of nutritional supplements, physical activity; the fourth level was coffee intake volume per day. The dependent variable in the model was self-reported type-2 diabetes (modelled with the binary response of higher prevalence—in this case ‘no’ respondents). The independent variables which remained in the final model were age (in years), marital status (single, married and other), diabetes mellitus and dyslipidaemia antecedents (yes or no), cigarette smoking (smoker, ex-smoker, non-smoker), use of supplement (yes or no), body mass index and coffee intake (≤100 mL, 101 to 400 mL, and more than 400 mL per day). Referent values in each level were arbitrarily established and based on previously published research [11,17]. Descriptive values were presented as raw values, while the results from the hierarchical linear regression model and Poisson regression for the PR were presented as weighted values.

## 3. Results

Of the 1,440 interviews, there were 78% (1,126) subjects contacted via residential telephone and 22% (314) by mobile telephone calls. The interviewed population ranged in age from 18 to 89 years and the majority were women (67%). Regarding coffee drinking habits, the vast majority were regular consumers (81%) and 65% reported drinking up to 400 mL of coffee per day. Table 1 shows frequency of coffee intake as a function of demographic, socio-behavioural, and BMI characteristics. Individuals were more likely to drink coffee at an older age and when married; coffee drinkers were also less physically active and more were smokers. Among smokers the number of cigarettes was higher in heavy coffee drinkers and lower in non-coffee drinkers. Overweight was present in 40% of the subjects and the overall mean BMI was 24.4 ± 4 kg/m^2^. Among non-coffee drinkers there were a high percentage of thin (26%) and normal BMI (21%) subjects. On the other hand, subjects consuming >400 mL/day of coffee had high percentages of overweight (19%) and obesity (22%). Sugar was the main form of sweetener used (74%) but was not quantified. In many circumstances the amount of sugar consumed with coffee is added to large containers (frequently a thermos) for family or general consumption.

Type-2 diabetes and family antecedents of NCCD in relation to coffee intake are presented in Table 2. Coffee consumers of >100 mL/day reported more cases of cancer in their parents (Table 2).

Because of the disproportionate number of females (more than statistically present in the population) we made adjustments for sex as well as for age and years of education, as these variables were shown to be biased in our population [25]. Results from the models refer to weighted values and reflect a corrected proportion of each variable in the study; thus, describing overall prevalence ratio for the association of coffee intake and diabetes, controlling for confounders observed in Table 1.

In univariate analysis, it was found that being single (PR = 1.08; p = 0.002), younger (PR = 0.99; p < 0.001), smoking (PR = 1.03; p = 0.006) and having a lower BMI (PR = 0.99; p = 0.008), had a higher prevalence of not having type-2 diabetes when compared to the proposed alternatives (Table 3).

In the multivariate analyses after adjusting confounding variables (Table 3): younger (PR = 0.99; p < 0.001), use of multivitamins and/or polyminerals (PR = 1.03; p = 0.037) and moderate coffee intake (101 to 400 mL/day) (PR = 1.03; p = 0.039) had a higher prevalence of not having diabetes mellitus compared to their referent categories. In other words, moderate coffee drinkers (100 to 400 mL/day) had an almost 3% higher prevalence of not having diabetes mellitus than those drinking ≤ 100 mL/day. However, for high coffee consumers (>400 mL/day), the prevalence of not having diabetes mellitus was not statistically different from those who consume less than 100 mL/day.

One particular coffee brand was used by 80% of the interviewed participants. Coffee preparation was mainly filtered (82%), while 5.2% preferred to consume instant coffee and 2.8% consumed either filtered or instant coffee. Additionally, we measured the content of chlorogenic acids (CGA’s) and caffeine in the most reported coffee brand, using an HPLC method by a respected coffee laboratory in Brazil. On a 100 g powder basis there were 837 mg of CGA’s and 1,461 mg of caffeine. Coffee dilution recommended and widely used in Brazil is 10 g of coffee powder to 100 mL of hot water [26].

## 4. Discussion

This study suggests that moderate coffee consumption is protective against type-2 diabetes occurrence in urban Brazilians living in the Federal District. Because the association was only significant at intermediate level of coffee intake and not at lower or higher levels, it indicates other intervening variables. Nevertheless, our findings are in agreement with other cross-sectional studies done in Greece [18], Sweden [14,27], Spain [28], the Netherlands [16,29], Denmark [30], Finland [31] and Japan [32]. In these European countries coffee is most likely consumed in instant form (or soluble) and as an infusion (or paper filtered). It should be noted that not only the method of coffee preparation but also the amount of coffee consumed and attendant components are measured and modelled in ways that render studies complex to compare. A comprehensive comparison of parameters in cross-sectional studies is shown in Table 4. It is worth mentioning that this study is consistent with the bulk of high quality epidemiology done with prospective studies [2,17].

Notably, in our study, demographic and environmental variables most likely to interact with coffee consumption were age, years of schooling, sedentarism, cigarette smoking, and nutritional status. In this regard, van Dam and Feskens [9] observed high coffee intake among those with lower schooling levels and among younger subjects [16]. However, regarding cigarette smoking several studies have also shown a significant and positive association with coffee intake [9,11,15,16]. Regarding physical activity, Salvaggio *et al.* [33], found a positive relation between coffee intake and physical activity.

This was a descriptive study designed to evaluate self-reported coffee consumption, life style and prevalence of type-2 diabetes. There are a number of potential limitations that should be considered when interpreting the results of this study. The research was conducted by telephone interview, which precluded individuals with no access to telephone lines. The sampling protocol adequately represented individuals from all regions of the Federal District but cannot be taken as representative of the entire population. It is also known that a cross-sectional study involves attendant limitations. The causality or temporality of the relationships between coffee intake and diabetes cannot be determined.

We are aware that in this type of study we cannot properly address the required information concerning other confounding factors associated with type-2 diabetes, nor can we deal with variables directly related to coffee preparations and consumption. Nevertheless, compared to published cross-sectional studies, our data add a unique characteristic from a developing country with a nutritional lifestyle that is quite different from that of developed countries (Table 4). Therefore, this study permits health scientists to contextualize a persistent association of coffee consumption and prevalence of type-2 diabetes.

It should be noted that our sample size, comparatively, is among the 50% largest of the published studies (Table 4). Although there are several metabolic active substances in the coffee beverage that might interfere with pathophysiology of type-2 diabetes, so far caffeine is the most studied and pharmacologically described one. In this regard, our study is one of the few that adds information pertaining to caffeine intake *per se*. Furthermore, the amount of sugar taken with coffee may vary with the quality of the product consumed but also with the habitual ‘sweet tooth’ that is culturally developed. The fact that we found a protective effect against type-2 diabetes occurrence in moderate coffee consumers may reflect a threshold related to added sugar in high coffee consumers (>400 mL).

Excessive sugar added to beverages may increase the risk of NCCD [34,35]. However, there are no studies in Brazil that quantify the amount of sugar added to coffee. In a clinical trial Louie *et al.* [36] showed that only sweetened black coffee significantly reduces postprandial glycaemia when compared with either black coffee or hot water 1 hour prior to a high carbohydrate meal. In contrast, Sartorelli *et al.* [37] showed that addition of sugar to coffee had no difference in the protection against the risk of diabetes among French women.

Therefore there are still paradoxical findings from clinical and observational studies involving sweetened coffee and the risk of diabetes. A limitation of the present study is that sugar added to coffee was not quantified.

Multivariate methods are important tools because they allow determination of the relative roles of multiple factors. Hierarchical multivariate models go further and make it possible to include variables in the model at different levels of the causal chain with the sequential introduction of variables from the distal to the proximal level, following a prior conceptual model. The interpretation of conceptual models is complex and alternative explanations are often possible, depending on the order in which these factors are defined in the causal chain [38]. In this respect it should be noted that use of nutritional supplements and moderate coffee intake are associated with protection for type-2 diabetes after controlling for first and second levels, and for first, second and third levels factors hierarchically in our model, respectively. Because vitamin C is known to be one of the most used supplements in the population [39] (in our study it was the most used—data not shown) and as vitamin C has antioxidant properties [40], the association of moderate coffee persists after controlling of this factor.

Mainly as a result of the generation of compounds deriving from the Maillard reaction, the roasting process leads to substantial changes in the chemical composition and biological activities of coffee [41]. In addition to this, coffee consumption around the world varies greatly, thus causing an attendant variation in components as a result of bean-roasting and/or brewing technique [2]. In Brazil, medium-roast coffee is the most widely used [26]. A roasting time of 10 minutes (medium-dark roast) was found to produce coffee with optimal oxygen scavenging and chain breaking activities *in vitro* [42]. Del Castillo *et al.* [43] confirmed that light roast or medium roast coffee has a significantly higher antioxidant activity *in vitro* than green coffee. This difference was observed despite a 19% and 45% decrease in the chlorogenic acids (CGA) content in light and medium-roast coffee, respectively; this implies that other compounds make significant contributions to the total antioxidant activity of roasted coffee. Farah *et al.* [44] determined CGA content in different varieties of coffee. Total CGA content in regular green arabica samples was 5.1, 5.4, 6.4, and 5.6 g%, on a dry matter basis (dm), for Bourbon, Sumatra from Mandelim, Sumatra from Sulawesi, and Heirloom, respectively. The average levels of total CGA after 6, 7, and 8 minute of roasting were 3.4, 2.0, and 1.0 g% for regular coffee and 3.3, 1.8, and 0.9 g% for decaffeinated coffee (dm), respectively. Information on coffee composition from cross-sectional studies is rare. In Table 4, we present the amount of caffeine and CGA recalculated for 100mL of coffee from the few available data. Compared to other studies, our sample drank coffee with medium CGA content and the highest caffeine content, which may partially explain the absence of association with type-2 diabetes for high coffee consumers [4].

Overall, as summarized in Table 4, consistency of cross-sectional studies (like ours) from different countries underscores a biological mechanism associated with coffee composition. Among proposed components are minerals (magnesium) and phytochemicals (mainly chlorogenic acids) that *per se* or in combination could provide a unique antioxidant functionality that would benefit coffee drinkers [2,45]. Additionally, time of drinking coffee could also play a distinct role in glucose metabolism [37]. This adds a physiological component to the understanding of some benefits of coffee consumption.

## 5. Conclusions

These data complement the gradually growing body of information that provides evidence for the overall beneficial effects of coffee consumption in relation to type-2 diabetes. However, the biological effects of coffee constituents and other intervening constitutional and environmental variables deserve further investigation.

## Figures and Tables

**Table 1.** Population characteristics (socio-behavioural and body mass index) according to coffee intake groups from individuals in the Federal District, Brazil, 2006–2009.

**Characteristics**	**Coffee intake (mL/d)**								**P value⌖**

	**NCC**		**≤100**		**101–400**		**>400**		

**Total group (N)**	271		440		491		238		

	**N**	**%**	**N**	**%**	**N**	**%**	**N**	**%**	
**Age (years)**									
**<30**	106	26	153	38	108	26	40	10	**0.000**
**30–40**	81	20	127	31	143	34	63	15	
**40–60**	65	15	105	24	171	39	98	22	
**>60**	19	11	55	31	68	38	37	21	

**Years of education**									
**≤8**	56	15	116	31	127	34	76	20	
**8–11**	121	21	187	33	182	32	76	14	
**11–15**	74	19	111	29	140	36	63	16	0.055
**15–20**	13	16	22	27	29	35	18	22	

**Sex**									
**Male**	94	20	135	29	151	32	91	19	0.143
**Female**	177	18	305	32	340	35	147	15	

**Marital status**									
**Bachelor**	139	25	194	35	163	30	57	10	
**Married**	87	14	186	31	214	35	121	20	**0.000**
**Divorced**	21	19	24	21	42	38	25	22	
**Widow**	11	15	19	26	32	44	11	15	
**Co-habiting**	3	7	11	24	17	38	14	31	

**Physical activity**									
**Practiced**	165	22	229	30	245	33	111	15	**0.007**
**Non-practiced**	106	15	211	31	246	36	127	18	

**Minutes per week**									
**<150**	28	17	58	34	62	36	22	13	0.120
**150–300**	63	20	98	32	98	31	52	17	
**>300**	70	27	69	27	83	33	35	13	

**Use of supplements**									
**Yes**	24	18	41	31	49	38	17	13	0.674
**No**	247	19	399	30	442	34	221	17	

**Alcohol use**									
**Yes**	101	17	190	31	208	34	108	18	0.270
**No**	170	20	250	30	283	34	130	16	

**Alcohol intake (mL/day)**									
**≤100**	42	16	73	28	99	39	43	17	0.531
**100–199**	10	11	28	32	30	34	19	22	
**≥200**	19	18	36	34	31	30	19	18	

**Cigarette smoking**									
**Smoker**	15	9	37	21	65	37	59	33	0.000
**Ex-smoker**	31	12	67	27	102	41	48	19	
**Non-smoker**	225	22	337	33	324	32	131	13	

**Cigarettes (units/day)**									
**≤10**	12	12	28	27	37	36	26	25	0.007
**≥10**	3	4	9	13	24	35	33	48	

**BMI classes**									
**Undernutrition**	14	26	23	44	13	24	3	6	
**Eutrophic**	166	21	245	31	262	33	115	15	**0.002**
**Overweight**	56	14	123	30	154	37	79	19	
**Obesity**	23	18	34	27	42	33	28	22	

Notes: NCC = non-coffee consumers; N = number of subjects; % = percentage, BMI = body mass index (Kg/m^2^). Sixty participants did not state their weight and/or height for BMI calculations.

⌖ P value obtained by Fisher exact test with significance at p < 0.05.

**Table 2.** Raw frequencies for diabetes and family non-communicable diseases according to coffee intake in the Federal District, Brazil, 2006–2009.

**Knowledge of diseases**		**Coffee intake (mL per day)**								

		**NCC**		**≤100**		**100–400**		**>400**		***P value***^b^

		N	%	N	%	N	%	N	%	

		**Subjects**								
**Diabetes mellitus**	Yes	7	13	16	30	17	31	14	26	0.24
	No	263	19	421	31	470	34	216	16	
**Parent’s antecedents^a^**										
Hypertension (n = 1,317)	Yes	126	17	223	30	260	35	132	18	0.062
	No	130	23	174	30	184	32	88	15	

Dyslipidaemias (n = 1,127)	Yes	76	18	129	30	149	35	75	17	0.349
	No	153	22	210	30	224	32	111	16	

Diabetes mellitus (n = 1,332)	Yes	39	16	73	28	103	39	45	17	0.187
	No	217	20	329	31	351	33	175	16	

Cancer (n = 1,363)	Yes	23	13	49	27	70	39	39	21	0.016
	No	240	20	365	31	392	33	185	16	

Parkinson’s disease (n = 1,361)	Yes	8	38	3	14	9	43	1	5	0.056
	No	252	19	406	30	455	34	222	17	

Alzheimer’s disease (n = 1,361)	Yes	5	17	8	26	9	31	8	26	0.538
	No	259	20	402	30	455	34	215	16	

NCC = non-coffee consumers; N = number of subjects. Individuals who did not state their knowledge of diseases are not included in this table.

a Family antecedents: YES = father and/or mother, NO = without disease.

b P value obtained by the Fisher exact test with significance at p < 0.05.

**Table 3.** Hierarchical regression model for self-reported type-2 diabetes in individuals from the Federal District, Brazil, 2006–2009.*

**Variable**	**Unadjusted prevalence ratio (CI)**	**P value**	**Adjusted prevalence ratio (CI)**	**P value**
**First level**				
**Marital status**				
*Bachelor*	1.082 (1.028 to 1.138)	**0.002**	1.040 (0.996 to 1.086)	0.077
*Married*	1.044 (0.987 to 1.104)	0.131	1.044 (0.992 to 1.098)	0.094
*Other*	1 (referent)		1 (referent)	
**Age (years)**	0.997 (0.995 to 0.998)	**<0.001**	0.997 (0.996 to 0.999)	**<0.001**

**Second level**				
**Diabetes antecedents^a^**				
*Yes*	1 (referent)		1 (referent)	
*No*	1.033 (0.997 to 1.070)	0.073	1.010 (0.976 to 1.045)	0.582
**Dyslipidaemias antecedents^b^**				
*Yes*	1 (referent)		1 (referent)	
*No*	1.023 (0.997 to 1.050)	0.078	1.010 (0.985 to 1.034)	0.435

**Third level**				
**Smoking**				
*Smoker*	1.026 (1.007 to 1.046)	0.006	1.016 (0.995 to 1.039)	0.136
*Ex-smoker*	1.003 (0.975 to 1.032)	0.829	1.032 (0.997 to 1.068)	0.071
*Non-smoker*	1 (referent)		1 (referent)	
**Supplementation^c^**				
*Yes*	1.015 (0.993 to 1.037)	0.193	1.027 (1.001 to 1.053)	0.037
*No*	1 (referent)		1 (referent)	
**BMI (kg/m^2^)**	0.993 (0.988 to 0.998)	0.008	0.996 (0.992 to 1.00)	0.113

**Fourth level**				
**Coffee intake**				
*≤100 mL*	1 (referent)		1 (referent)	
*101–400 mL*	1.014 (0.989 to 1.039)	0.283	1.027 (1.001 to 1.053)	0.039
*>400 mL*	0.982 (0.945 to 1.021)	0.368	1.010 (0.967 to 1.054)	0.659

* Type-2 diabetes was modelled with the binary response of higher prevalence–96% of ‘no’ respondents. CI = confidence interval; BMI = body mass index (Kg/m^2^). 95% confidence interval.

a Diabetes antecedents: parents of the interviewed subjects with type-2 diabetes diagnosis.

b Antecedents of dyslipidaemias: parents of the interviewed subjects with diagnosis of hypercholesterolaemia and/or hypertriglyceridaemia.

c Supplementation: user or non-user of multivitamins and/or polyminerals.

Model was run after sample weighting for sex, age and years of study according to the year 2000 Brazilian census. Significant when P value was <0.05.

**Table 4.** Description of cross-sectional studies on coffee intake and type-2 diabetes.

**Reference**	**Country and Sample characteristics (n, sex, age)**	**Model used and results presentation**	**Observed results**					**Cup volume (mL)**	**Total CGA’s content (mg/100 mL)**	**Caffeine content (mg/100 mL)**
			**0 or <1 cup**	**1–2 cups or ≤2 cups**	**3–4 cups**	**>4 cups or 5–6 cups**	**>7 cups**			
Machado *et al*. (Present study)	BRAZIL *N* = 1,440 Both sexes 18 to 89 year	Hierarchical regression model and Poisson regression (PR for diabetes status)	1.0	1.027 (2.7% self report of ‘no’ diabetes)		1.010		120	84	146
Panagiotakos *et al.*^[18 ]^	GREECE *n* = 937 Both sexes 65 to 100 year	Multiple logistic regression analysis (multi-adjusted OR)	1.0	0.47		1.05		150	NI	28
Agardh *et al.*^[14]^	SWEDEN *N* = 7,949 Both sexes 35 to 56 year	Multiple logistic regression analysis (OR referred to as RR).	Men: 1.0 Women: 1.0		0.52 0.41	0.36 0.28		150–200	NI	67
Yamaji *et al.*^[15]^	JAPAN *N* = 3,224 Only males 46 to 59 year	Multiple logistic regression analysis	1.0	0.8	0.7	0.7		150	NI	40
Van Dam *et al.*^[16]^	NETHERLANDS *N* = 1,312 Both sexes 50 to 74 year	Adjusted differences in 2-hour glucose concentrations according to categories of coffee consumption using analysis of covariance. Logistic regression analysis	Baseline data: ≤2 cups/day multivariate-adjusted 2-hour post-load glucose concentrations were observed:		−0.52 mM	−0.76 mM	−0.87 mM	125	100	NI
Van Dam *et al.*^[ 29]^	NETHERLANDS *N* = 419 Only males 69 to 94 year	Multivariate models						NI	NI	NI
		Prevalence of glucose intolerance	29%		21%	14%				
		Multivariate-adjusted	1.0		0.63	0.35				

**Reference**	**Country and Sample characteristic s (n, sex, age)**	**Model used and results presentation**	**Observed results**					**Cup volume (mL)**	**Total CGA’s content (mg/100 mL)**	**Caffeine content (mg/100 mL)**

Bidel ^[31]^ (Academic dissertation)	FINLAND *n* = 2,956 Both sexes 45 to 64 year	Multiple logistic regression analysis	Coffee consumption as a continuous variable showed that an increment of one cup of coffee per day was associated with a 10% lower risk of impaired fasting glucose, an 8% lower risk of isolated impaired glucose tolerance, a 9% lower risk of impaired glucose regulation, and an 11% lower risk of hyperinsulinaemia if both men and women were combined.					240	35–175	42
Ärnlöv *et al.*^[27]^	SWEDEN *N* = 936 Only males 50 to 88 year	Multivariate regression models	An increase of one cup of coffee per day was associated with 0.16-units higher insulin sensitivity (insulin sensitivity index was determined by hyperinsulinaemic euglycaemic clamp).					150	NI	NI
Soriguer *et al.*^[28]^	SPAIN *N* = 1,226 Both sexes Adults (age not informed)	Multiple logistic regression analysis	Persons who drank coffee at least once per day had a lower risk for diabetes mellitus and impaired glucose tolerance (odds ratio, 0.66 [95% CI, 0.48 to 0.92]; P < 0.02) than persons who drank coffee only occasionally.					NI	NI	NI
Isogawa *et al.*^[32]^	JAPAN *N* = 4,620 Both sexes 40 to 50 year	NI	Coffee intake was inversely associated with the prevalence of fasting hyperglycaemia. Risk of having prevalent fasting hyperglycaemia: OR = 0.614					NI	NI	NI

Abbrevations: NI, not informed; PR, prevalence ratio; OR, odds ratio; RR, relative risk; CGA’s, chlorogenic acids.
